# Vγ9Vδ2 T cells expressing a BCMA—Specific chimeric antigen receptor inhibit multiple myeloma xenograft growth

**DOI:** 10.1371/journal.pone.0267475

**Published:** 2022-06-16

**Authors:** Xi Zhang, Yu Yang Ng, Zhicheng Du, Zhendong Li, Can Chen, Lin Xiao, Wee Joo Chng, Shu Wang

**Affiliations:** 1 Department of Biological Sciences, National University of Singapore, Singapore, Singapore; 2 Department of Haematology-Oncology, National University Cancer Institute Singapore, National University Health System, Singapore, Singapore; 3 Cancer Science Institute of Singapore, National University of Singapore, Singapore, Singapore; 4 Department of Medicine, Yong Loo Lin School of Medicine, National University of Singapore, Singapore, Singapore; Alfred I DuPont Hospital for Children, UNITED STATES

## Abstract

Vγ9Vδ2 T cells are immune effector cells capable of killing multiple myeloma (MM) cells and have been tested in clinical trials to treat MM patients. To enhance the MM cell killing function of Vγ9Vδ2 T cells, we introduced a BCMA-specific CAR into *ex vivo* expanded Vγ9Vδ2 T cells through electroporation of the CAR-encoding mRNA. The modified Vγ9Vδ2 T cells displayed a high cytolytic activity against BCMA-expressing MM cell lines *in vitro*, while sparing BCMA-negative cells, including normal B cells and monocytes. Subsequently, we intravenously injected KMS-11 human MM cells to generate a xenograft mouse model. The treatment of the tumor-bearing mice with Zometa and anti-BCMA CAR- Vγ9Vδ2 T cells resulted in a significant reduction of tumor burden in the femur region, as well as the overall tumor burden. In association with the decrease in tumor burden, the survival of the MM cell-inoculated mice was markedly prolonged. Considering the potential of Vγ9Vδ2 T cells to be used as off-the-shelf products, the modification of these cells with a BCMA-specific CAR could be an attractive option for cancer immunotherapy against bone marrow cancer MM.

## Introduction

Vγ9Vδ2 T cells are part of the innate immunity system that are well-suited for adoptive cell therapy (ACT) for cancer and can exert potent effector functions to directly kill target cells in a non-MHC restricted fashion, being less prone to alloreactivity and less likely to cause graft-versus host-disease (GvHD) when used in allogeneic setting [1, 2]. Critically for ACT against cancer, Vγ9Vδ2 T cells can home in various tissues and infiltrate into a range of human malignancies as an important component of tumor-infiltrating lymphocytes (TILs) [1, 2]. Moreover, Vγ9Vδ2 T cells can act as antigen-presenting cells by taking up tumor antigens and cross-presenting the processed peptides to induce αβ T-cell activation, thus further stimulating systematic anti-tumor immune responses after locally destroying tumor cells [1, 2]. The multifaceted immune functions of Vγ9Vδ2 T cells make them good effector candidates for ACT.

Multiple myeloma (MM) is one of the most common hematological malignancies characterized by the uncontrolled proliferation of monoclonal plasma cells in the bone marrow [3, 4]. Clinical studies with γδ T cells were initiated following an observation published in 1999 reporting the increased numbers of peripheral blood γδ T cells in patients with MM treated with aminobisphosphonates for bone resorption [5]. Studies afterwards have established that peripheral blood γδ T cells expanded by aminobisphosphonate zoledronic acid can target primary MM cells and MM-inducing bone-resorbing osteoclasts, thus inhibiting the vicious cycle of tumor progression and bone destruction [6–12]. Based on these findings, adoptive transfer of Vγ9Vδ2 T cells *ex vivo* expanded with zoledronic acid was performed in a pilot clinical trial. The treatment resulted in a measurable increase in the number of the effector cells in the peripheral blood and bone marrow without serious treatment-related adverse effects, although clinical outcomes were limited to the remained serum M-protein levels at baseline in four of the six tested patients with MM [13]. These clinical findings indicate that there is still substantial room to improve the efficacy of ACT with Vγ9Vδ2 T cells.

ACT of chimeric antigen receptor (CAR)-modified alpha beta T cells specifically recognizing tumor-associated antigens has emerged as a promising novel treatment for hematologic malignancies [14]. Two recent publications have demonstrated the impressive efficacy of CAR-T cell therapy designed to target B-cell maturation antigen (BCMA) that is universally expressed in malignant plasma cells [15, 16]. In these studies, the objective response rate (ORR) was ranging from 88% to 95.5% in heavily pre-treated MM patients. However, in spite of the promising early responses, MM eventually relapsed in the majority of the treated patients [17, 18]. The anti-MM therapy with BCMA-specific CAR T cells was also associated with severe cytokine release syndrome as well as neurotoxicity in nearly all patients [19–21]. To address the issues in CAR-T cell therapy for MM, especially those related to the access and safety, allogeneic “off-the-shelf” CAR cell products and mRNA transfected CAR-T cells are under development [22].

Considering the attractive immune functions of Vγ9Vδ2 T cells and their potential in treating MM as discussed above, we generated BCMA-CAR-modified Vγ9Vδ2 T cells through mRNA electroporation and investigated the feasibility of using them to control MM progression in a mouse xenograft model.

## Results

### Generation of Vγ9Vδ2 T cells expressing anti-BCMA mRNA CAR

**Fig 1:**
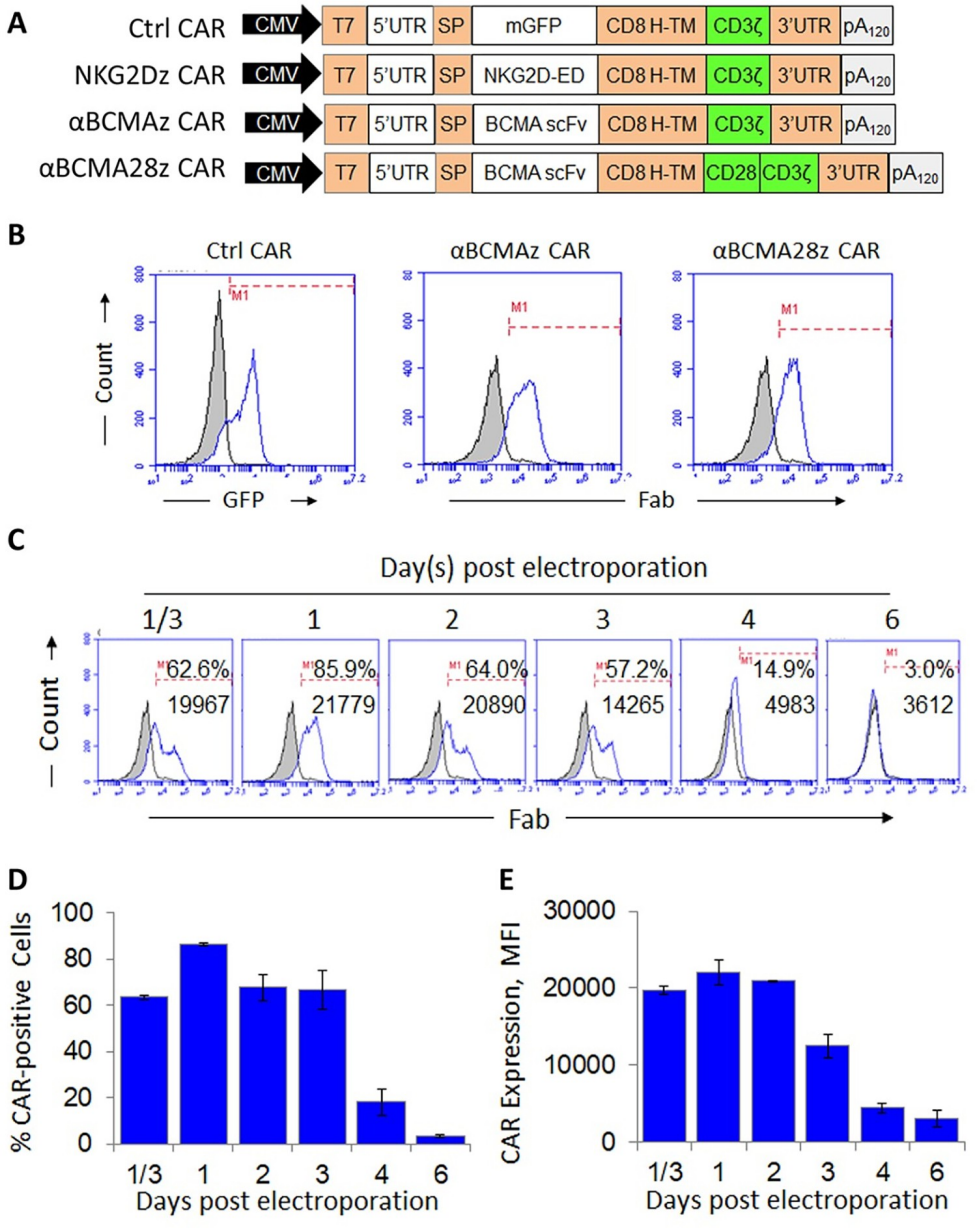
CARs used in this study and CAR expression on Vγ9Vδ2 T cells. (**A**) Schematic diagrams of the plasmid constructs used for CAR mRNA production. The 1^st^ and 2^nd^ generation αBCMA CARs were tested. A 1^st^ generation NKG2D CAR was included for comparison. The mGFP Ctrl CAR was constructed by replacing the scFv fragment of αBCMA CAR with the EGFP coding sequence. The plasmid templates of the CARs were PCR amplified using a CMV forward primer and a reverse primer with 150 Ts. The PCR amplicons were then used for RNA transcription to generate mRNA molecules encoding the CARs for the electroporation of Vγ9Vδ2 T cells. (**B**) Flow cytometric analysis to demonstrate the expression of αBCMA CARs and the Ctrl CAR on Vγ9Vδ2 T cells. Black lines represent wild-type Vγ9Vδ2 T cells stained with an isotype control antibody. Blue lines represent Vγ9Vδ2 T cells electroporated with a CAR construct and stained with the anti-Fab antibody. Cell samples were collected 24 hours post-electroporation for staining. The results of one representative experiment out of three independent experiments with three different donors are shown. (**C**) Time lapse analysis of αBCMAz CAR expression after electroporation. Vγ9Vδ2 T cell samples were collected at the indicated time points for staining. Black lines represent wild-type Vγ9Vδ2 T cells stained with an isotype control antibody. Blue lines represent CAR Vγ9Vδ2 T cells stained with an anti-Fab antibody to show the CAR expression. Percentage and mean fluorescence intensity (MFI) of cells positive for CAR staining over isotype controls are indicated in each histogram. The results of one representative experiment out of three independent experiments with three different donors are shown. (**D**) The mean ± SD of the percentage (*Left*) and MFI (*Right*) of cells expressing αBCMAz CAR. The results were obtained from three independent experiments with three different blood donors.

To redirect Vγ9Vδ2 T cell specificity towards MM cells with RNA CAR approach, we first constructed two plasmid vectors serving as templates for anti-BCMA mRNA CAR synthesis. We designed both 1^st^ and 2^nd^ generation anti-BCMA CAR constructs with the same BCMA-specific scFv (C11D5.3) fused in-frame to the human CD8 hinge and CD8 transmembrane domain (**Fig 1A**). The scFv C11D5.3 was chosen due to its high binding affinity to BCMA as previously reported [23, 24]. For the 1^st^ generation CAR, the CD3ζ chain signaling domain was fused directly downstream the CD8 transmembrane domain, named αBCMAz. In the 2^nd^ generation CAR construct, a CD28 costimulatory domain was fused in frame between the CD8 transmembrane domain and the CD3ζ signaling domain, named αBCMA28z. The control construct was generated by replacing the anti-BCMA scFv sequence with the EGFP coding sequence. A 1^st^ generation NKG2D CAR, NKG2Dz, constructed in our previous study [25] was included for CAR activity comparison.

Healthy donor-derived Vγ9Vδ2 T cells were generated and electroporated with mRNA encoding different CARs as described in our previous study [25, 26]. The transfection efficiency was evaluated with an antibody against GFP for GFP expression or anti-mouse F(ab’)_2_ for Fab expression and the efficiencies between 70% to 86% were observed (**Fig 1B**). Time-lapse analysis of the αBCMAz CAR expression was performed at 8 hours, 1, 2, 3, 4, 6 day(s) post electroporation (**Fig 1C**). The percentage of Fab-expressing cells and the mean fluorescence intensity (MFI) value of Fab staining both peaked on day 1 post electroporation (percentage up to 86%, MFI above 20 000), decreased gradually over the next three days, and became hardly detectable by day 6 (**Fig 1D**). This self-limited mRNA CAR expression provides a useful platform for fast evaluation of CAR constructs and immediate toxicity screening in a clinical trial.

### Evaluation of in vitro cytolytic activity of CAR-modified Vγ9Vδ2 T cells

**Fig 2:**
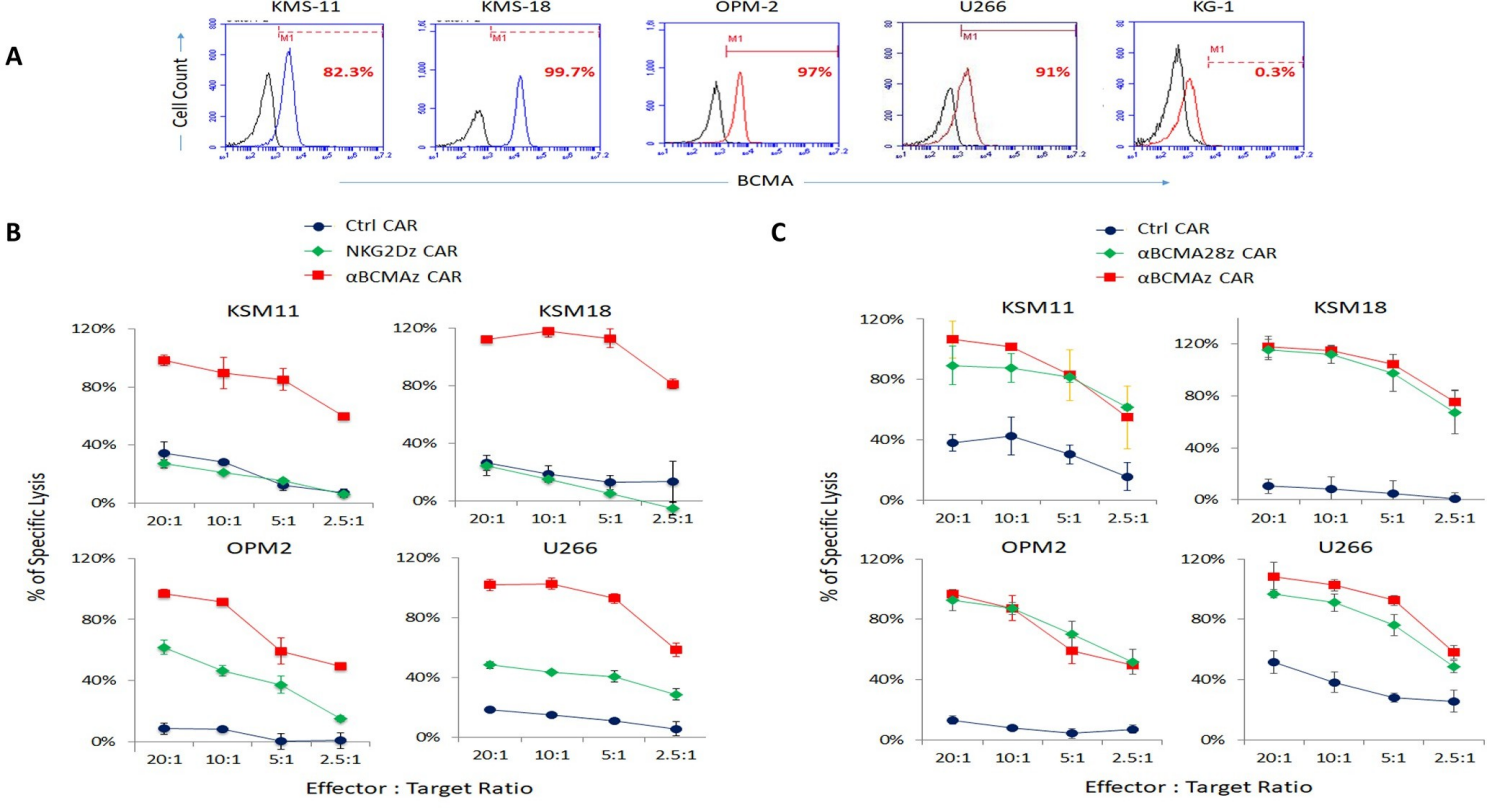
Tumor cell lysis induced by Vγ9Vδ2 T cells modified with NKG2D CAR and αBCMA CARs. DELFLA^®^ EuTDA cytotoxicity assay (3 hours EuTDA culturing) was used to assess tumor cell lysis efficiency. (**A**) BCMA expression on the tested tumor cell lines. BCMA-positive KMS-11, KMS-18, OPM-2 and U266 human MM cell lines and BCMA-negative KG-1 cell line were tested in this study. (**B**) Comparison between NKG2Dz CAR and αBCMAz CAR. (**C**) Comparison between αBCMAz CAR and αBCMA28z CAR. The results of one representative experiment out of at least three independent experiments with different donors are shown in B and C.

As previously reported, BCMA is uniformly expressed on the malignant plasma cells of many MM patients while its expression is hardly detectable on normal human tissues or hematopoietic stem cells [17, 23, 27]. Here we detected high levels of BCMA expression in established human MM cell lines including KMS-11 (82%), KMS-18 (100%), OPM-2 (97%), and U266 (91%), while the acute myelogenous leukemia cell line KG-1 was BCMA-negative (**Fig 2A**).

We then used a short-term (3 hours) DELFLA^®^ EuTDA assay to evaluate *in vitro* cytotoxic effects of CAR-modified Vγ9Vδ2 T cells on these MM cells. We recently published a paper demonstrating that NKG2D CAR can harness the tumor killing power of Vγ9Vδ2 T cells against several tumor cell lines [25]. Since the tested MM cell lines also express NKG2D ligands (S1 Fig), we first compared a 1^st^ generation NKG2D CAR (NKG2Dz CAR) and the 1^st^ generation αBCMA CAR (αBCMAz CAR) after introducing them into Vγ9Vδ2 T cells. **Fig 2B** showed that αBCMAz CAR-modified Vγ9Vδ2 T cells exhibited much more potent *in vitro* tumor cell lysis activity against all 4 tested MM cell lines than NKG2Dz CAR-modified Vγ9Vδ2 T cells. We further compared *in vitro* cytotoxic activities of αBCMAz CAR and the 2^nd^ generation αBCMA CAR (αBCMA28z CAR). While the two CARs showed similar potencies in increasing the tumor cell lysing activity of Vγ9Vδ2 T cells against KMS-18 and OPM-2, we observed that αBCMAz-expressing Vγ9Vδ2 T cells exhibited higher *in vitro* lytic activity towards KMS-11 and U266 tumor cells compared to αBCMA28z CAR-modified Vγ9Vδ2 T cells (**Fig 2C**). This result is consistent with our previous study with Vγ9Vδ2 T cells modified with different NKG2D CARs, in which the 1^st^ generation of NKG2D RNA CAR displayed a superior tumor cell cytolytic activity as compared to the 2^nd^ and 3^rd^ generation RNA CARs in a short-term cytotoxicity assay, possibly because mRNA encoding the 1st generation CAR is smaller in size [25].

### αBCMA CAR-expressing Vγ9Vδ2 T cells mediate antigen-specific cytotoxic immune responses

**Fig 3:**
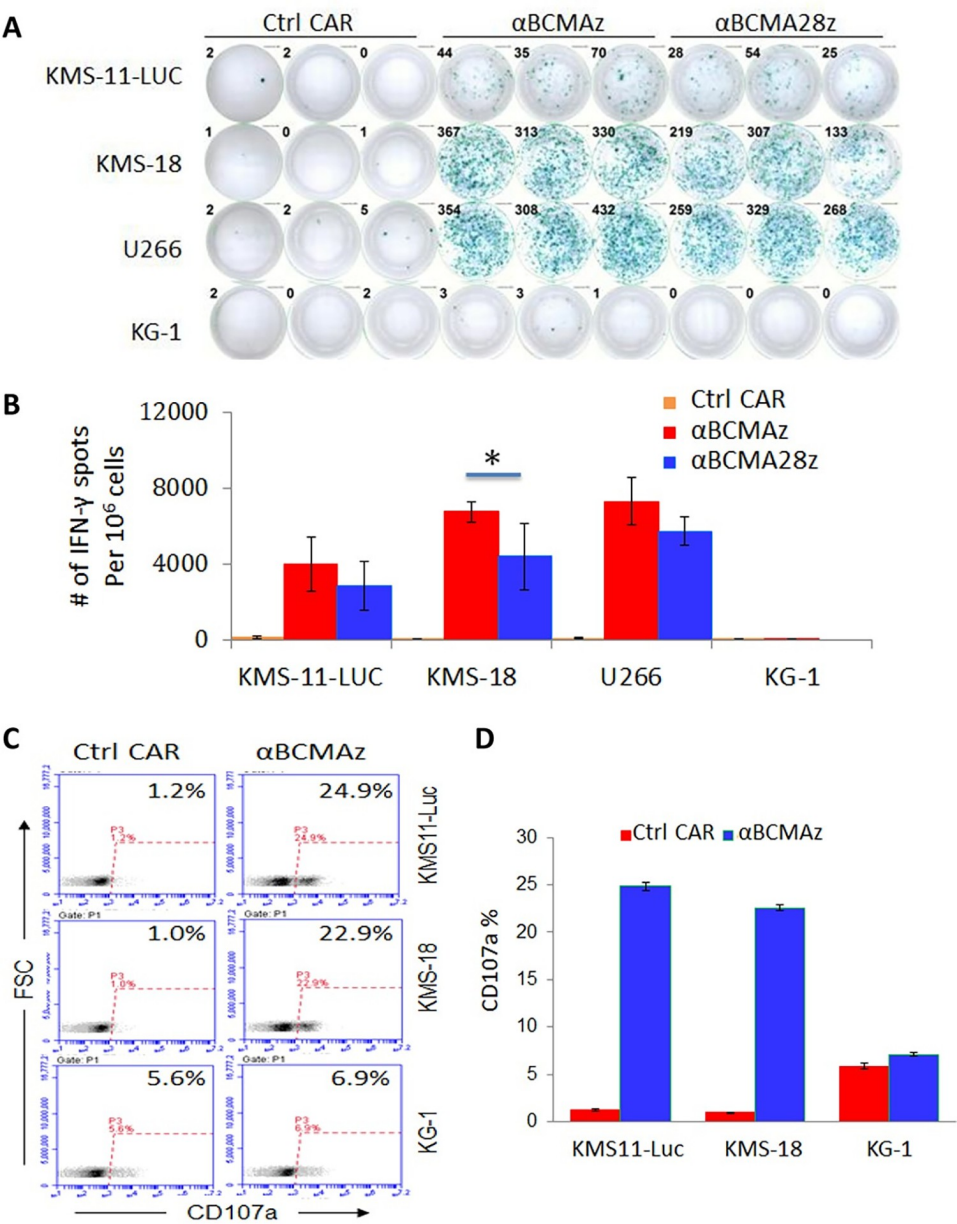
Antigen-specific cytotoxic immune responses of Vγ9Vδ2 T cells after αBCMA CAR modification. (**A**) IFN-γ secretion as determined by an IFN-γ ELISPOT assay. CAR-modified Vγ9Vδ2 T cells were co-cultured with BCMA-positive KMS-11, KMS-18 and U266 tumor cells as well as BCMA-negative KG-1 tumor cells at an E:T ratio of 5:1 overnight before the assay. Ctrl CAR-transfected T cells served as negative controls. The images of the increased IFN-γ secretion triggered by tumor antigen-specific recognition of αBCMAz CARs are shown. (**B**) Bar graphs to show mean IFN-γ spots per 1 × 10^6^ Vγ9Vδ2 T cells ± SD from triplicate cultures. (**C**) The degranulation of αBCMAz CAR-modified Vγ9Vδ2 T cells upon activation by BCMA-positive and BCMA-negative tumor cells. After co-culturing with the tumor cells, Vγ9Vδ2 T cells were gated and analyzed for CD107a expression by flow cytometry. Dot plots of CD107a are shown. Data are representative of three independent experiments with three different blood donors. (**D**) Bar graphs to show the mean percentages of Vγ9Vδ2 T cells expressing CD107a. The results shown represent mean ± SD of three independent experiments with three different donors.

We proceeded to evaluate the antigen-specific immune responses of αBCMA CAR-expressing Vγ9Vδ2 T cells with the IFN-γ ELISPOT assay, given that Th-1 cytokine IFN-γ secretion is associated with antitumor activity of Vγ9Vδ2 T cells [28]. To this end, Vγ9Vδ2 T cells transfected with αBCMA CARs were co-cultured with various target cell lines at an E:T ratio of 5:1 for 24 hours before measuring IFN-γ release. The scanned ELISPOT plates of BCMA-positive tumor cell lines KMS-11, KMS-18, and U266 showed that while there were almost no spots after the co-culturing with the control Vγ9Vδ2 T cells transfected with mGFP RNA CAR, high numbers of IFN-γ-positive spots were observed with BCMA-specific CAR-equipped Vγ9Vδ2 T cells (**Fig 3A**). Correspondingly, the analysis of the plates with a spot-counting machine indicated that the number of positive spots for anti-BCMA CAR Vγ9Vδ2 T cells was significantly higher than that for the control Vγ9Vδ2 T cells (p < 0.001, **Fig 3B**). The quantitative analysis further demonstrated that αBCMAz CAR was more potent than αBCMA28z CAR in stimulating IFN-γ secretion. When the BCMA-negative cell line KG-1 was tested, the CAR-T cell treatment induced just few numbers of spots. The results obtained from this ELISPOT assay suggested that the αBCMA CAR-expressing Vγ9Vδ2 T cells stimulated IFN-γ secretion in an antigen-specific manner.

We next investigated the degranulation of αBCMAz CAR-modified Vγ9Vδ2 T cells upon activation by MM cell lines, KMS-11 and KMS-18. The BCMA-negative cell line KG-1 was again included as the negative control. To this end, CAR-modified Vγ9Vδ2 T cells were co-cultured with the tumor cell lines for 6 hours and the upregulation of CD107a, as a marker of degranulation, was evaluated with flow cytometry (**Fig 3C and 3D**). Cell surface mobilization of CD107a, closed to 25%, was observed upon BCMA activation of αBCMAz CAR-modified Vγ9Vδ2 T cells, whereas no appreciable degranulation was seen in the mGFP CAR control group. Meanwhile, CD107a was not up-regulated when the CAR expressing Vγ9Vδ2 T cells were incubated with the control cell line KG-1, indicating this degranulation was specifically mediated by BCMA activation. Putting together, these *in vitro* findings demonstrated Vγ9Vδ2 T cells could mediate antigen-specific cytotoxic immune responses after modification with αBCMA CARs.

### αBCMA CAR-expressing Vγ9Vδ2 T cells control tumor growth in vivo

**Fig 4:**
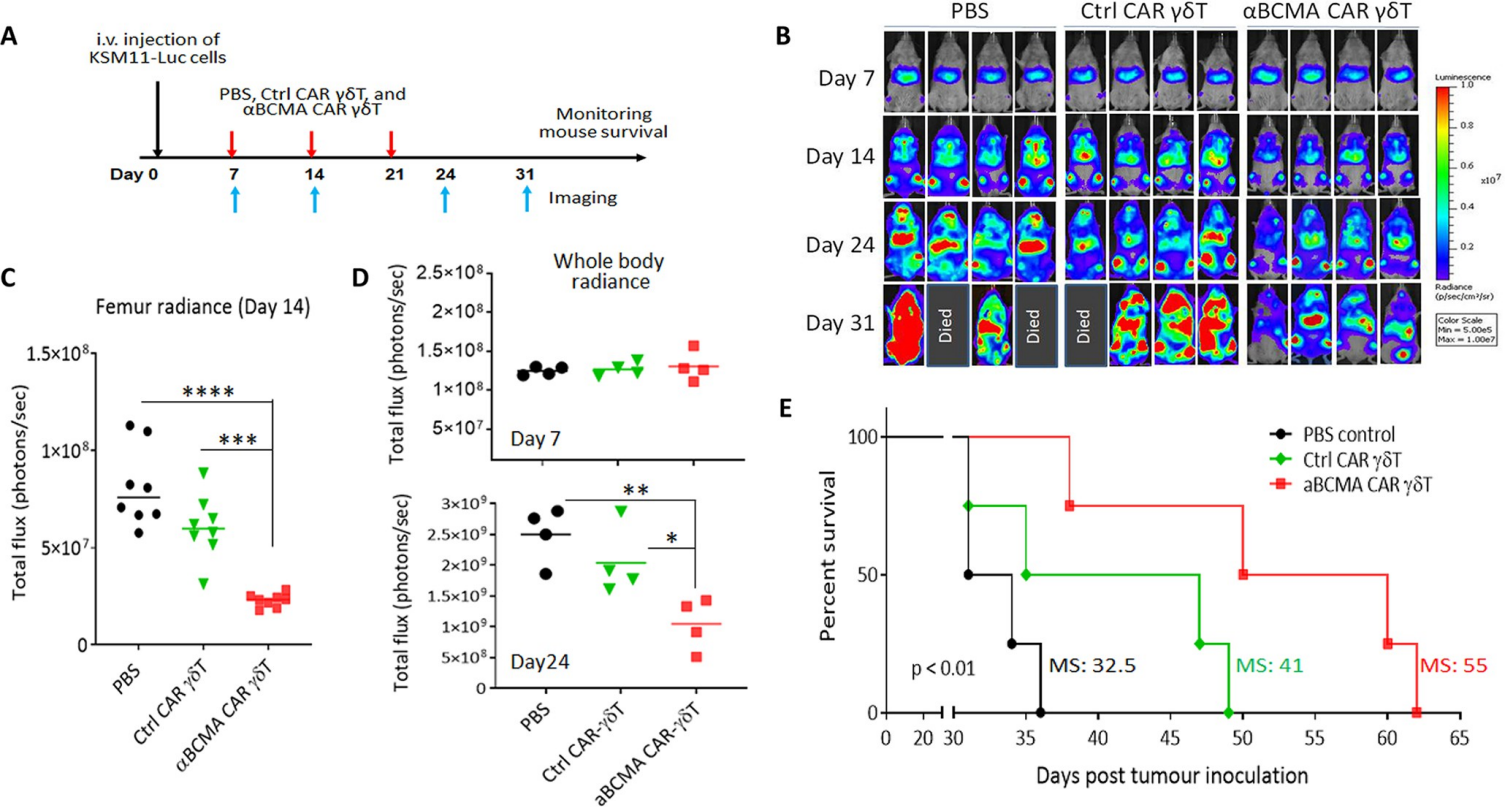
Effects of anti-BCMA CAR-modified Vγ9Vδ2 T cells in a KMS-11 multiple myeloma (MM) mouse model. (**A**) Experiment design. NSG mice were γ-irradiated (300 cGy). One day later, 5 x 10^6^ KMS11-Luc cells were i.v. injected through the lateral tail vein. Started at day 7 post-tumor inoculation, three groups of mice (n = 4 per group) were treated with PBS, Ctrl CAR Vγ9Vδ2 T cells or αBCMAz CAR Vγ9Vδ2 T cells once a week for three weeks, with 1×10^7^ Vγ9Vδ2 T cells in 100 μl PBS per injection. All mice were i.p. injected with 2 μg of Zometa 24 hours before the treatments. (**B**) Growth of KMS-11 xenografts was monitored by bioluminescent imaging on the indicated days. Bioluminescent images are shown. Three mice in the PBS and Ctrl CAR γδT cell groups died on day 31. (**C**) Bioluminescence flux values in the upper end of the femur on Day 14 from each mouse of respective groups were plotted. ***, ****: p < 0.001 and 0.0001, respectively. (**D**) Bioluminescence flux values acquired via whole body BLI on Day 7 and Day 24 from each mouse of respective groups were plotted. *, **: p < 0.05 and 0.01, respectively. (E) Survival of mice was monitored up to Day 62 post tumour inoculation and analyzed by the Kaplan-Meier method. The median survival (MS) of each group is indicated in days.

We then assessed the *in vivo* efficacy of CAR-modified Vγ9Vδ2 T cells. Based on the *in vitro* results shown above, αBCMAz CAR was selected for *in vivo* testing. A xenogeneic mouse model of MM was established by the intravenous injection of a KMS-11-Luc cell line that stably expresses the firefly luciferase reporter gene (S2 Fig). Seven days after tumor cell inoculation, mice were randomized into three groups (n = 4 per group) and treated with the intravenous injection of PBS, Vγ9Vδ2 T cells expressing the Ctrl CAR, or Vγ9Vδ2 T cells expressing αBCMAz CAR. The treatments were performed once a week for three weeks (**Fig 4A**). To sensitize KMS-11 MM cells and promote Vγ9Vδ2 cell proliferation, all mice were intraperitoneally (i.p.) injected with 2 μg of Zometa 24 hours before the treatments.

Around 2 weeks after the tumor cell injection, a non-invasive whole-body bioluminescent imaging (BLI) demonstrated the orthotropic engraftment of the MM cells in the bone marrow (**Fig 4B**), as well as the engraftment in other areas outside of the bone marrow (Fig 4B), similar to what observed when MM progresses to later stages. Meanwhile, we found that the tumor burden in the mice treated with the anti-BCMA CAR Vγ9Vδ2 T cells were obviously reduced on day 14 post-tumor inoculation as compared to that in the PBS- or the Ctrl CAR-treated groups (Fig 4B). A quantitative analysis of the tumor luciferase signals in the upper end of the femur (n = 8 femora from 4 mice) on day 14 indicated that the treatment with anti-BCMA CAR-expressing Vγ9Vδ2 T cells was highly effectively in controlling MM growth in the bone marrow: the luciferase radiant efficiency ([p/sec/cm^2^/sr]/[μW/cm^2^]) in the femora decreased from 8.1 x 10^7^ in the mice receiving PBS and 6.0 x 10^7^ in the mice receiving Ctrl CAR- Vγ9Vδ2 T cells to 2.3 x 10^7^ following the treatment with anti-BCMA CAR-expressing Vγ9Vδ2 T cells (p < 0.001 and 0.0001, respectively, **Fig 4C**). Changes in the luciferase signals in the whole body from day 7 to day 14 further confirmed that the inhibition of tumor growth by the anti-BCMA CAR treatment (**Fig 4D**). More importantly, the median survival time increased from 32.5 and 41 in the PBS and Ctrl CAR Vγ9Vδ2 T cell groups, respectively, to 55 days in the anti-BCMA CAR Vγ9Vδ2 T cell group (p = 0.0023, **Fig 4E**). Notably, all mice treated with Vγ9Vδ2 T cells did not display typical GVHD symptoms thar caused by allogenic CAR-αβ T cells infusion, such as significant weight loss, arched back, ruffled fur and diarrhea. Thus, the anti-BCMA CAR Vγ9Vδ2 T cell treatment prolonged median survival by 69% and 34%, respectively, as compared to the PBS and Ctrl CAR groups, significantly delaying disease progression in MM tumor-bearing mice.

## Discussion

The current BCMA-specific CAR cell therapy mostly uses autologous αβ T cells from patients. Given that such an individualized manufacturing process is costly, labor-intensive, and can be logistically challenging, allogeneic CAR-T cell products are generated from blood cells of healthy donors using gene editing and developed as “off-the-shelf” therapeutics [29]. Vγ9Vδ2 T cells could be a potential cell type of choice that can be used to generate “off-the-shelf” CAR cell products without gene editing. These innate-like T lymphocytes share many phenotypical and functional characteristics of both innate and adaptive immunity, and are known to be non-alloreactive (no MHC class I/ II restriction) and do not induce GvHD during allogeneic Vγ9Vδ2 T cell therapies [30].

The activation of Vγ9Vδ2 T cells is usually associated with lower secretion levels of cytokines such as IL-2, IFNγ, and TNF, as compared to the activation of αβ T cells [30]. Thus, the use of Vγ9Vδ2 T cells can hypothetically reduce the risk of causing undesired side effects associated with the use of highly active CAR- αβ T cells, most notably the cytokine release syndrome (CRS). A previous study has shown that CAR-modified Vγ9Vδ2 T cells display a cytolytic activity similar to that of αβ T cells modified with the same CAR but a lower level of cytokine secretion [31], suggesting the possible superiority of CAR-Vγ9Vδ2 T cell therapy in risk mitigation for CRS.

Efficient gene transfer into Vγ9Vδ2 T cells represents a practical challenge to the generation of CAR-expressing Vγ9Vδ2 T cells. To establish a proof-of-concept that CAR modification of Vγ9Vδ2 T cells can enhance their anti-myeloma activity, we used a non-integrating, mRNA electroporation technology to express anti-BCMA CAR in *ex vivo* expanded Vγ9Vδ2 T cells, which generated short-lived CAR-expressing cells. Understandably, transient CAR expression necessitates multiple infusions of a relatively higher dose of CAR-modified immune effector cells to achieve antitumor effects. As an attractive feature of the technology, the duration and potency of CAR effects can be controlled by different dosing and infusion schemes, providing convenience in controlling CAR toxicity. Through discontinuing the infusion of CAR-Vγ9Vδ2 T cells, an excessive response caused by the toxicity related to recognition of normal tissues and/or cytokine storms can be stopped. This could be practically important, as it has been shown that clinical trials with anti-BCMA CAR-T cells are associated with characteristic toxicities including severe CRS and neurotoxicity [19, 20].

Several antigens have been explored for CAR-T cell-based immunotherapy against MM. Among them, BCMA represents one of the most attractive targets given its universal overexpression in malignant plasma cells and restrictive expression pattern in B-cell lineage cells [17, 23, 27]. This expression pattern is different from many other tested antigens, such as CS1, CD38 and CD56, that are expressed also in normal hematopoietic cells. Another interesting feature for BCMA antigen is that the anti-BCMA CAR-T cells could be activated by both low and high BCMA expressing-tumour cells and shown to be similarly efficient in killing them. In an open-label, multiple centres phase 1 clinical trial for treating relapsed or refractory MM with anti-BCMA CAR-T cells, the high clinical response rate was observed to be similar for both patients have less than 50% and more than 50% BCMA antigen expression level, which was consistent with their pervious *in vitro* results in killing a broad range of BCMA expressing tumour cell lines [15]. For our current study, we found that both αBCMAz and αBCMA28z CAR-modified Vγ9Vδ2 T cells displayed robust *in vitro* killing efficacy towards all BCMA-expressing cell lines of high but varied expression level (range from 82% - 100%). Since the researchers conducted the clinical trials did not reveal the specific sequences of the scFv region for their CAR construct and they used autologous αβ T cells in treating the patients, we could not conclude that our anti-BCMA CAR-modified Vγ9Vδ2 T cells would be functional exactly as theirs. However, this is definitely a valuable point to be investigate in the future *in vitro* and *in vivo* studies.

Although Vγ9Vδ2 T cells have been well recognized for their potential in treating the bone marrow cancer MM [5, 13], the impact of these cells as immune effector cells in CAR cell therapy against MM has not been evaluated before. Given the fact that the lytic bone disease is a hallmark of MM [4], we first confirmed that adoptively transferred Vγ9Vδ2 T cells could efficiently localize to the bone. The quick and long-term accumulation of the immune effector cells in the bone represents a favorable feature for the treatment of bone lesions (data not shown). We then determined the effects of anti-BCMA CAR-modified Vγ9Vδ2 T on bone lesions associated with the intravenous injection of MM cells. This animal model displayed extensive vascular metastases and fast progressive tumor growth, appearing to be therapeutically challenging. Although the treatment with the CAR-T cells was not sufficient to eradicate the established tumors or induce tumor regression, it resulted in obviously retarded tumor growth and enhanced survival in tumor-bearing mice. As the main finding of the study, we reported that these CAR-T cells were effective in reducing tumor burden in the upper end of the femur in our MM mouse model (Fig 4B & 4C). Our finding, for the first time, provides clear experimental evidence that Vγ9Vδ2 T cells modified with a CAR can result in notable therapeutic outcomes for MM bone lesions.

In conclusion, the current study demonstrated that anti-BCMA CAR-modified Vγ9Vδ2 T cells are myeloma-reactive *in vitro* and *in vivo*, suggesting them as a potential candidate for tumor immunotherapy against MM. Since Vγ9Vδ2 T cells are ultrasensitive to immunosuppressive tumor microenvironment in the bone marrow of MM patients [32–34], the development and assessment of combination therapies that using immune checkpoint blockade agents to amplify the specific antitumor response of CAR-Vγ9Vδ2 T cells is warranted in future studies [35].

## Materials and methods

### Generation of CAR-Vγ9Vδ2 T cells

Human PBMCs were isolated from fresh buffy coats to expand Vγ9Vδ2 T cells. After 7 days of Zometa treatment, cells were mixed with γ-irradiated K562 Clone A aAPCs at an immune cell: K562 ratio of 1:100 for co-culturing. K562 Clone A aAPCs expressing CD64, CD86 and CD137L were described before [26, 36]. After 10 days of co-culturing, the cells were harvested for experiments.

The scFv sequence (C11D5.3) used for the anti-BCMA CAR was described previously [23]. The DNA fragment from the scFv was cloned in frame with CD8 hinge-CD8 transmembrane-CD3zeta in the pFastBac plasmid under the control of CMV and T7 promoters. The capped mRNA was synthesized by *in vitro* transcription of the purified DNA template for electroporation of Vγ9Vδ2 T cells.

### In vitro experiments

Target cancer cells were cultured as per manufacturer’s instructions. Flow cytometric analysis was performed with Accuri C6 cytometer (BD Biosciences, Franklin Lakes, NJ). The cytolytic activity of CAR-modified NK cells was examined with a non-radioactive method (DELFIA EuTDA Cytotoxicity Reagents kit, PerkinElmer, Waltham, MA).

## Animal experiments

A mouse xenograft model of human MM was established by intravenous injection of 5 x 10^6^ KMS-11-Luc cells through the tail vein. To evaluate the therapeutic effects of CAR-Vγ9Vδ2 T cells, these CAR-T cells were intravenously injected into the tumor-bearing mice and tumor progression was monitored by luminescent imaging.

All handling and care of animals was performed according to the guidelines for the Care and Use of Animals for Scientific Purposes issued by the National Advisory Committee for Laboratory Animal Research, Singapore. The animal study protocol was reviewed and approved by Institutional Animal Care and Use Committee (IACUC), the Biological Resource Centre, the Agency for Science, Technology and Research (A*STAR), Singapore (Permit Number: BRC IACUC 140930).

Behaviors and survival of the mice were monitored closely. Humane endpoints were used and mice were euthanized when moribund by placing them in the induction chamber filled with CO2 from compressed gas cylinder. The flow rate for CO2 euthanasia systems was displaced as 30% to 70% of the chamber volume/min under IACUC guideline. Gas flow was maintained for at least 1 minute after apparent clinical death observed. To confirm euthanasia, cervical dislocation was performed.

### Statistical analysis

Data are presented as mean ± standard deviation (SD). All statistics were performed with GraphPad Prism 7 (San Diego, CA, USA). P values < 0.05 were considered significant.

For the details of Materials and Methods, see S1 File.

## Supporting information

S1 Fig Expression of NKG2D ligands on MM tumor cell lines. The expression of MICA, MICB, ULBP1, ULBP2, ULBP3, ULBP4, ULBP5 and ULBP6 was assessed by flow cytometry with respective antibodies.
(PDF)

S2 Fig Generation of KSM-11-Luc cell lines. KMS 11 cells were transfected to stably express the firefly luciferase reporter gene and EGFP reporter gene under the control of human cytomegalovirus promoter, followed by EGFP sorting selection. (A) EGFP expression in the mixed cell population, which was used for EGFP sorting and single cell cloning. (B) In vitro luciferase activity assay. Among the 4 single cell clones were tested, 3 of them, Clones 1, 2 and 3 are positive. Clone 1 was used for the animal experiment.
(PDF)

S1 File Supplemental materials and methods. (DOCX)
