# Supplementary material for: Vγ9Vδ2 T cells expressing a BCMA—Specific chimeric antigen receptor inhibit multiple myeloma xenograft growth
Source: PLoS One. 2022 Jun 16;17(6):e0267475. doi: 10.1371/journal.pone.0267475 (PMC9202950; doi:10.1371/journal.pone.0267475)
Supplement: S1 Fig — The expression of MICA, MICB, ULBP1, ULBP2, ULBP3, ULBP4, ULBP5 and ULBP6 was assessed by flow cytometry with respective antibodies. (PDF) [file pone.0267475.s001.pdf]

Supplemental Figure 1

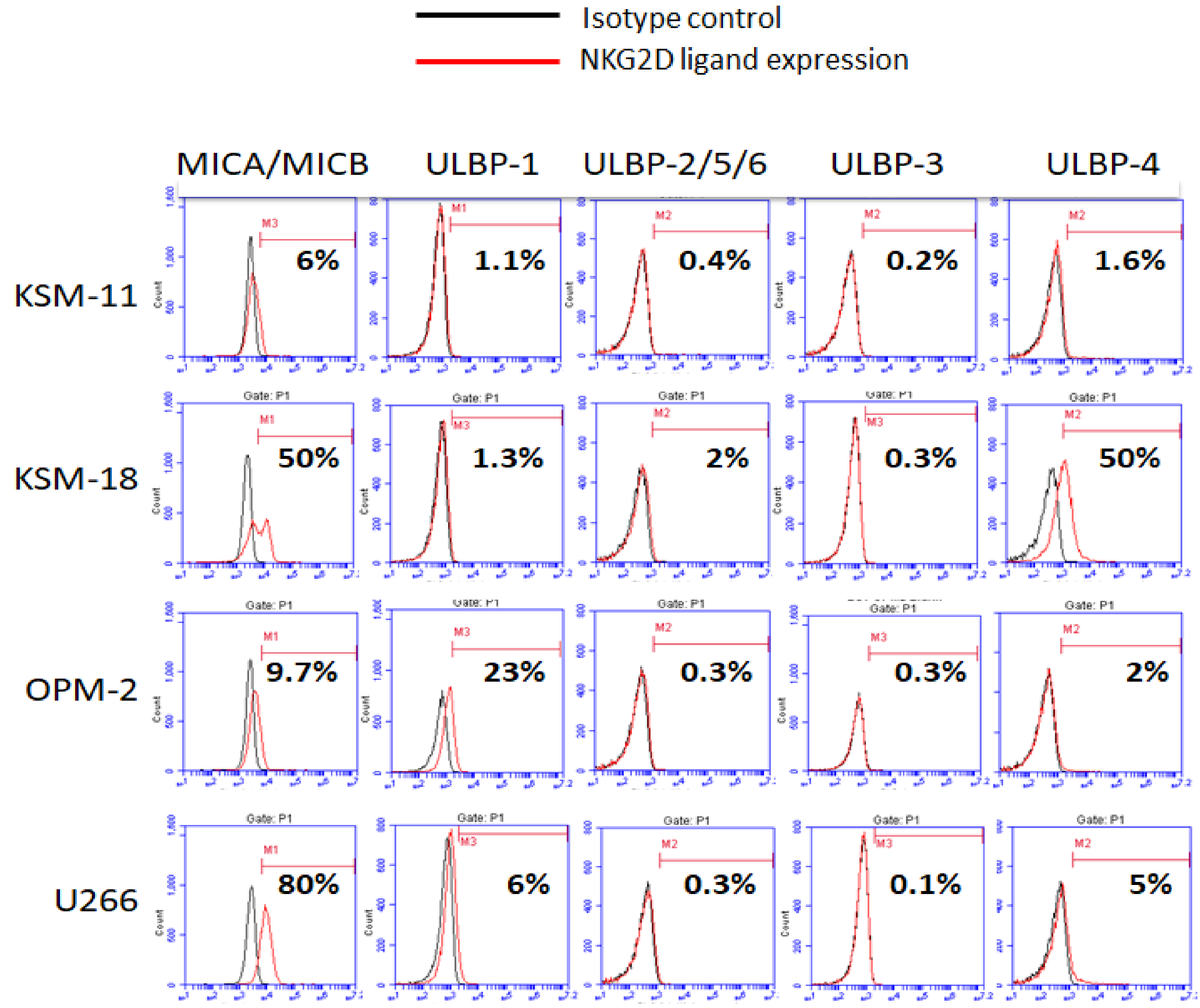

Supplemental Figure 1. Expression of NKG2D ligands on MM tumor cell lines. The expression of MICA, MICB, ULBP1, ULBP2, ULBP3, ULBP4, ULBP5 and ULBP6 was assessed by flow cytometry with respective antibodies.
