# Supplementary material for: Vγ9Vδ2 T cells expressing a BCMA—Specific chimeric antigen receptor inhibit multiple myeloma xenograft growth
Source: PLoS One. 2022 Jun 16;17(6):e0267475. doi: 10.1371/journal.pone.0267475 (PMC9202950; doi:10.1371/journal.pone.0267475)
Supplement: S2 Fig — KMS 11 cells were transfected to stably express the firefly luciferase reporter gene and EGFP reporter gene under the control of human cytomegalovirus promoter, followed by EGFP sorting selection. (A) EGFP expression in the mixed cell population, which was used for EGFP sorting and single cell cloning. (B) In vitro luciferase activity assay. Among the 4 single cell clones were tested, 3 of them, Clones 1, 2 and 3 are positive. Clone 1 was used for the animal experiment. (PDF) [file pone.0267475.s002.pdf]

Supplemental Figure 2

A

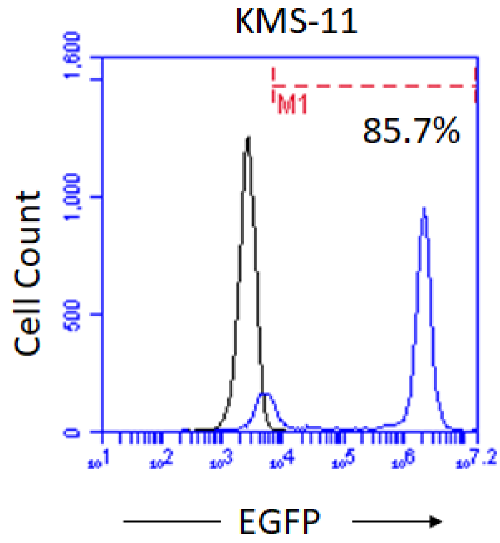

B

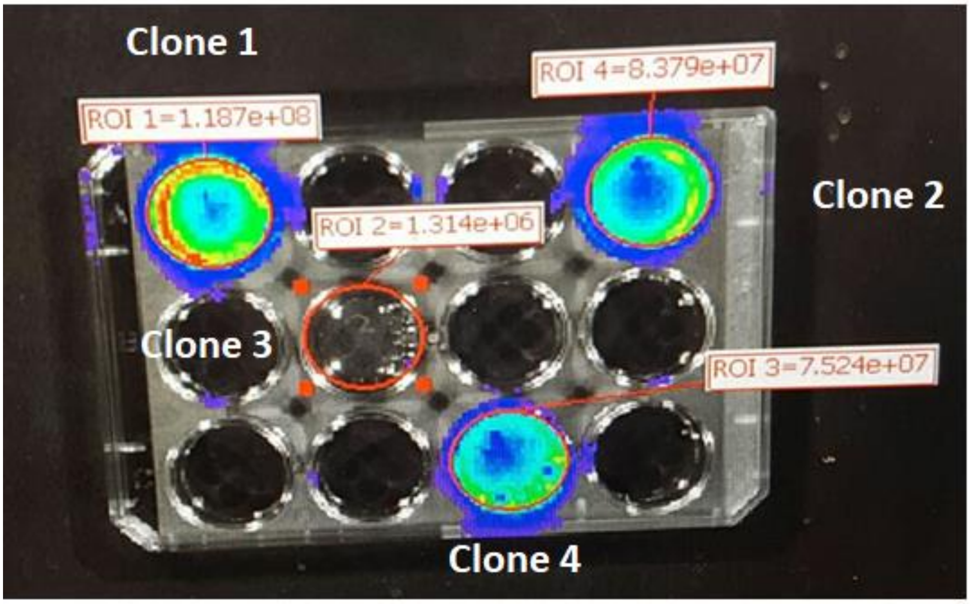

Supplemental Figure 2. Generation of KSM11-Luc cell lines. KMS-11 cells were transfected to stably express the firefly luciferase reporter gene and EGFP reporter gene under the control of human cytomegalovirus promoter, followed by EGFP sorting selection. (**A**) EGFP expression in the mixed cell population, which was used for EGFP sorting and single cell cloning. (**B**) In vitro luciferase activity assay. Among the 4 single cell clones were tested, 3 of them, Clones 1, 2 and 3 are positive. Clone 1 was used for the animal experiment.
