# Supplementary material for: Vγ9Vδ2 T cells expressing a BCMA—Specific chimeric antigen receptor inhibit multiple myeloma xenograft growth
Source: PLoS One. 2022 Jun 16;17(6):e0267475. doi: 10.1371/journal.pone.0267475 (PMC9202950; doi:10.1371/journal.pone.0267475)
Supplement: S1 File — (DOCX) [file pone.0267475.s003.docx]

**Vγ9Vδ2 T Cells Expressing a BCMA‐specific Chimeric Antigen Receptor Inhibit Multiple Myeloma Xenograft Growth**

**SUPPLEMENTAL INFORMATION**

**Materials and Methods**

***Cells and cell culture conditions***

Human MM cell lines KMS-11 (JCRB, Osaka, Japan), KMS-18 (JCRB, Osaka, Japan), OPM-2 (DSMZ, Braunschweig, Germany) and U266 (ATCC, Manassas, VA, USA) were maintained in RPMI (Lonza Biotech, Basel, Switzerland) supplemented with 10% heat-inactivated fetal bovine serum (FBS, Hyclone, Logan, UT, USA). Human acute myelogenous leukemia cell line KG-1 (ATCC, Manassas, VA, USA) was maintained in IMDM (Lonza Biotech) supplemented with 10% FBS. Human myelogenous leukemia cell line K562 (ATCC, Manassas, VA, USA) was maintained in IMDM supplemented with 10% FBS.

Buffy coats of healthy donors were collected from National University Hospital Singapore, Department of Laboratory Medicine Blood Transfusion Service, as approved by the institutional review board of National University of Singapore (NUS-IRB Reference Code B-14-133E). Human PBMCs were isolated from fresh buffy coats by density gradient centrifugation using Ficoll-Paque (GE Healthcare, Milwaukee, WI, USA). To expand Vγ9Vδ2 T cells, PBMCs (2 x 106) were seeded in a 24 well plate at day 0 and activated by 5 µM Zometa (Sigma-Aldrich, St Louis, MO, USA) in 1 ml AIM-V (Life Technologies, Carlsbad, CA) supplemented with 5% human AB serum (Valley Biomedical, Winchester, VA, USA), and 300 IU/ml human recombinant IL-2 (PeproTech, Rocky Hill, NJ, USA). After 7 days of Zometa treatment, cells were mixed with γ-irradiated K562 Clone A aAPCs at an immune cell: K562 ratio of 1:100 for co-culturing. K562 Clone A aAPCs expressing CD64, CD86 and CD137L were described before.26, 38 After 10 days of co-culturing, the cells were harvested for experiments.

***mRNA CAR preparation and electroporation of T cells***

The scFv sequence (C11D5.3) used for the anti-BCMA CAR was described previously 23. DNA fragments from the above scFv sequence were synthesized by Integrated DNA technologies (Singapore) and cloned in frame with CD8 hinge-CD8 transmembrane-CD3zeta in the pFastBac plasmid under the control of CMV and T7 promoters. The membrane-bound GFP (mGFP) CAR was produced by replacing the scFv sequence with the EGFP coding sequence. To generate DNA templates for in vitro mRNA synthesis, the DNA fragment from the CMV promoter to α-globin 3’ untranslated region of the modified pFastBac plasmid was PCR amplified with a synthesized 150-nt poly(A) tail added to the 3’ end and phenol-chloroform purified. The capped mRNA was synthesized by in vitro transcription of the purified DNA template using the mMESSAGE mMACHINE T7 ULTRA transcription kit (Invitrogen, Carlsbad, CA, USA) and stored at -80 °C.

For mRNA electroporation, 1 x 107 expanded Vγ9Vδ2 T cells were washed with PBS, re-suspended in 100 μl Opti-MEM (Life Technologies, Carlsbad, CA, USA) and mixed with 5 μg mRNA. The electroporation condition was optimized using 250 V, 4 ms and 1 pulse in the BTX Agilepulse in vivo waveform electroporation system (Harvard Apparatus, Holliston, MA, USA). Electroporated cells were immediately added to pre-warmed culture medium for overnight recovery.

***Flow cytometry and IFN-γ ELISPOT assay***

To detect the cell surface expression of mRNA CAR, aliquots of electroporated cells were stained by biotinylated goat anti-mouse F(ab’)2 antibody (Jackson Immunoresearch, West Grove, PA, USA) for one hour, followed by allophycocyanin (APC)-conjugated streptavidin staining (BD Biosciences, Franklin Lakes, NJ, USA) for 30 min. For phenotyping of immune cells, following anti-human antibodies were used: fluorescein isothiocyanate (FITC)-conjugated anti-Vδ2 TCR (Biolegend, San Diego, CA, USA), APC-conjugated anti-Vγ9 (Biolegend), APC-conjugated anti-CD19 (Biolegend) and PE-conjugated anti-CD14 (BD Biosciences). APC-conjugated anti-human BCMA antibody (Biolegend) was used for detecting BCMA expression. The staining procedure followed the manufacturer’s protocol strictly. All stained cells were washed and re-suspended in MACS buffer (Miltenyi Biotec, Bergisch Gladbach, Germany) and subjected to flow cytometric analysis with Accuri C6 flow cytometer (BD Biosciences). Cells stained with appropriate isotype control antibodies were used for population gating.

For flow cytometry analysis of CD107a expression, Vγ9Vδ2 T cells were co-cultured with target cells at an effector to target (E:T) ratio of 5:1 overnight at 37°C in the presence of GolgiStopTM (BD Biosciences) and PE-conjugated anti-CD107a antibody (BD Biosciences) or isotype matched control antibody and then analyzed.

To evaluate IFN-γ production, Vγ9Vδ2 T cells were stimulated by various target cells at an E:T ratio of 5:1 for 24 hours. Culture supernatants were collected and used for IFN-γ analysis with the ELISPOT kit (Mabtech, Nacka Strand, Sweden) according to the manufacturer’s protocol. The plates were read and analyzed by an ELISPOT scanner (CTL, Ltd., Cleveland, OH, USA).

***Cytotoxicity assay***

The cytolytic activity of CAR-expressing Vγ9Vδ2 T cells was examined with a non-radioactive method (DELFLA® EuTDA Cytotoxicity Reagents kit, PerkinElmer, Waltham, MA, USA). Time-resolved fluorescence was measured in Victor3TM multilabel plate counter (Perkin Elmer). The E:T ratios used ranged from 20:1 to 2.5:1. Control groups were set up to measure spontaneous release (only target cells added), maximum release (target cells added with 10 µl provided lysis buffer), and medium background (no cell added). Killing activity was calculated using the following formula:

$$\text{Specific Lysis=}\frac{\text{Experimental release (counts)-Spontaneous release (counts)}}{\text{Maximal release} \left( \text{counts} \right)\text{-Spontaneous release (counts)}}\text{×100\%}$$

***Animal experiment***

Immunodeficient non-obese diabetic/severe combined immunodeficiency/IL-2Rγcnull (NSG) mice (6-8 weeks old) were used in the current study. For an in vivo migration experiment, Vγ9Vδ2 T cells (5 × 106) were labelled with Xenolight DiR (Perkin Elmer) and injected via the tail vein (intravenous; i.v.) into three NSG mice. Cell distribution was monitored using an IVIS® spectrum imaging platform with Living Image software (Perkin Elmer) for 2 weeks. The animals were euthanized at the end of the experiment and femora were collected for ex vivo imaging.

For in vivo efficacy evaluation of CAR- Vγ9Vδ2 T cells, a mouse xenograft model of human MM was established by intravenously inoculating 5 x106 KMS-11-Luc cells 24 hours after γ-irradiation (300 cGy). To generate KMS11-Luc cells, KMS-11 cells were transfected to stably express the firefly luciferase reporter gene and EGFP reporter gene under the control of human cytomegalovirus promoter, followed by EGFP sorting selection (Fig. S2). On day 7 post-tumor inoculation, tumor engraftment was confirmed by live bioluminescence imaging (BLI) monitored using the IVIS® system. Mice with similar BLI signal intensity were randomly divided into different treatment groups (n = 4 per group) and i.v. injected with 1 × 107 Vγ9Vδ2 T cells in 100 μl PBS or the same volume of PBS without cells per injection, once a week for three weeks. Zometa (2 μg per mouse, Sigma-Aldrich, St. Louis, MO, USA) was intraperitoneally injected into mice 24 hours before the treatments. Tumor progression was monitored by BLI. Behaviors and survival of the mice were monitored closely. Humane endpoints were used and mice were euthanized when moribund.

All handling and care of animals was performed according to the guidelines for the Care and Use of Animals for Scientific Purposes issued by the National Advisory Committee for Laboratory Animal Research, Singapore. The animal study protocol was reviewed and approved by Institutional Animal Care and Use Committee (IACUC), the Biological Resource Centre, the Agency for Science, Technology and Research (A*STAR), Singapore (Permit Number: BRC IACUC 140930).

***Statistics***

Data are presented as mean ± standard deviation (SD). All statistics were performed GraphPad Prism 7 (San Diego, CA, USA). P values < 0.05 were considered significant.
